# Asking the Right Questions: How Early-Life Exposures Influence Later Development of Disease

**DOI:** 10.1289/ehp.120-a403a

**Published:** 2012-10-01

**Authors:** Julia R. Barrett

**Affiliations:** Julia R. Barrett, MS, ELS, a Madison, WI–based science writer and editor, has written for *EHP* since 1996. She is a member of the National Association of Science Writers and the Board of Editors in the Life Sciences.

Prenatal and early-life environmental insults ranging from malnutrition to toxic exposures can tilt the odds toward development of adverse health effects decades later. These effects likely occur, at least in part, through alterations in an individual’s genetic potential to thrive in the environment in which he or she will live—in other words, these early challenges set the bar for what’s “normal,” and the fetus and infant adapt for a less-than-optimal environment in ways that may contribute to adult-onset disease. A new review examines the evidence for an association between early-life exposures and later disease, and proposes an agenda for future research and risk assessment [*EHP* 120(10):1353–1361; Boekelheide et al.].

One of the most intensively studied instances of adult disease attributable to prenatal circumstances involves children of women who were pregnant during the Dutch famine of 1944–1945. At birth these individuals were small for gestational age, and as adults they had increased incidence of obesity, diabetes, and cardiovascular disease. Furthermore, their children also were small for gestational age, suggesting that such effects can be transmitted across generations, a finding replicated in animal studies of prenatal malnutrition and toxic exposure.

Human and animal studies now link a multitude of early-life hardships, including prematurity, low birth weight, maternal infection during pregnancy, toxic exposures, and malnutrition, to a wide array of adult-onset diseases. Timing of the insult appears to be an important factor in the consequences. In the Dutch cohort, for example, deprivation in early gestation was associated with coronary heart disease, hypertension, dyslipidemia, and obesity later in life, whereas mid-gestation deprivation was linked with obstructive airway disease and impaired glucose tolerance.

Many adverse outcomes associated with early-life events could be explained by epigenetic changes, in which the genes themselves are unaltered but their expression is persistently blocked, increased, or decreased by environmentally induced molecular tweaks to the components controlling transcription and translation. Such changes could occur directly to a developing fetus or indirectly by, for example, undermining placental function or triggering disease-susceptibility genes. Nonepigenetic changes are also possible, as illustrated by so-called obesogenic chemicals that can activate the key regulator of fat cell growth and development.

Many potential research avenues arise from the accumulating evidence for delayed effects of early-life exposures. Central themes include characterizing epigenetic and nonepigenetic changes that predict disorders, prioritizing investigations, and identifying critical time points with regard to susceptibility. Methods for collecting and analyzing the data alone constitute a research priority, while the ultimate goal is to enable accurate risk assessment. Both experimental evidence in animals and observational human data will be needed to assess causal relationships.

